# Highly sensitive diagnostic method for colorectal cancer using the ratio of free DNA fragments in serum

**DOI:** 10.20407/fmj.2017-025

**Published:** 2018-12-06

**Authors:** Satoshi Arakawa, Soji Ozawa, Takashi Ando, Hiroya Takeuchi, Yuko Kitagawa, Jin Kawase, Hisanori Oshima, Hidetoshi Nagata, Koji Atsuta, Rie Yoshida, Norihiko Kawabe, Shunji Umemoto, Zenichi Morise, Akihiko Horiguchi

**Affiliations:** 1 Department of Gastroenterological Surgery, Fujita Health University Bantane Hospital, Nagoya, Aichi, Japan; 2 Department of Gastroenterological Surgery, Tokai University School of Medicine, Isehara, Kanagawa, Japan; 3 Department of Surgery, School of Medicine, Keio University, Shinjyuku, Tokyo, Japan; 4 Department of Surgery, Hamamatsu University School of Medicine, Hamamatsu, Shizuoka, Japan; 5 Department of Surgery, Fujita Health University School of Medicine, Toyoake, Aichi, Japan

**Keywords:** Colorectal cancer, DNA fragments, Alu247/115 ratio, Real-time polymerase chain reaction

## Abstract

**Objectives:**

The correlations of the ratio of long-/short-chain DNA fragments in blood with the existence of cancer and the clinicopathological features of colorectal cancer (CRC) were examined. The potential use of this ratio for diagnostic screening was evaluated.

**Methods:**

DNA concentrations were amplified using Alu247 for long-chain DNA fragments and Alu115 for long- and short-chain DNA fragments. The Alu247/115 ratio was calculated for 60 patients with CRC and 24 healthy volunteers. The correlation of the Alu247/115 ratio with clinicopathological variables and the efficacy of this ratio as a tumor marker were examined. The Alu247/115 ratio cut-off value was set using a receiver operating characteristic (ROC) curve.

**Results:**

The Alu247/115 ratio was significantly higher in patients with CRC than in healthy volunteers (P<0.001). The Alu247/115 ratio was also significantly higher in patients with Dukes stage A or B CRC than in healthy volunteers (P=0.034) as well as in patients with Dukes C or D CRC than in those with Dukes A or B CRC (P=0.016). Among patients with CRC, the Alu247/115 ratio was significantly higher in those with than without venous invasion (P=0.031). Using the cut-off value set from the ROC curve, the sensitivity of the Alu247/115 ratio was significantly higher than that of the carcinoembryonic antigen level (P=0.004) or the carbohydrate antigen 19-9 level (P<0.001).

**Conclusion:**

Our data suggest that the Alu247/115 ratio is a promising tool for highly sensitive and early detection of CRC.

## Introduction

The incidence of colorectal cancer (CRC) is high worldwide and is increasing in Japan.^1–3^ The establishment of multidisciplinary treatment has improved the prognosis of patients with CRC.^4,5^ The Dukes and TNM classifications are important tools when making decisions regarding treatment of CRC, and these classifications are well-correlated with the post-treatment prognosis.^6^ Dukes stage A tumors are limited to the mucosal or submucosal layer, Dukes stage B tumors exhibit invasion through the muscularis propria but without lymph node and/or distant metastasis, Dukes stage C tumors involve lymph node metastasis, and Dukes stage D tumors involve distant metastasis.^7^ Theoretically, patients with Dukes stage A and B cancer have tumors within the local primary area, and those with Dukes stage C and D cancer have tumors that have spread outside the primary area. Pathological examination of resected specimens makes Dukes classification staging simpler than TNM staging; thus, the Dukes system is used to stratify CRC into groups of different prognoses and requirements for adjuvant radiotherapy and chemotherapy.^8^ However, local recurrence and multiorgan metastases after treatment remain problematic partly because of the difficulty in detecting small tumors at an early stage.

Carcinoembryonic antigen (CEA) and carbohydrate antigen 19-9 (CA19-9), the two major tumor markers for CRC, are not useful for detecting small tumors because of their low sensitivity. In contrast, detection of cancer-related DNA abnormalities in the peripheral blood could enable small CRCs to be detected at an earlier stage than with presently available tumor markers, fecal occult blood tests, or imaging modalities.^9,10^

DNA fragments originating from somatic cells exist in peripheral blood.^11^ Short-chain DNA fragments of 185 to 200 base pairs (bp) arising from apoptosis are mainly observed under normal conditions in the absence of disease.^12^ However, DNA fragments of ≥200 bp, known as long-chain DNA fragments, are observed in the blood of patients with malignancies.^13^ These fragments are derived from necrosis of ischemic tumor cells and the surrounding tissues that are injured by tumor progression. Several studies have focused on DNA fragments. Wang et al.^14^reported a higher percentage of long-chain DNA fragments in the blood of patients with gynecological and breast cancer. Umetani et al.^15,16^ established two new primers, Alu247 and Alu115, for the quantitative polymerase chain reaction (PCR) detection of long- and short-chain DNA fragments. They revealed a correlation between tumor progression and the ratio of long-/short-chain DNA fragments in the blood of patients with CRC and patients with breast cancer using quantitative PCR for the Alu sequence, the most common repeat sequence in the human genome.^15,16^ Thus, the Alu247/115 ratio (the ratio of DNA quantities amplified with Alu247 [long-chain DNA fragments]/DNA quantities amplified with Alu115 [long- and short-chain DNA fragments]) may be a promising biomarker for the screening and early detection of CRC.

This study was performed to examine the correlation of the Alu247/115 ratio with the existence of cancer and the clinicopathological features of patients with various stages of CRC and to evaluate the potential use of this ratio for diagnostic screening.

## Methods

Serum samples from 60 patients with CRC (male:female, 34:26; median age, 73.5 years; range, 50–90 years) and 24 healthy volunteers (male:female, 6:18; median age, 31.5 years; range, 23–54 years) were examined. Healthy volunteers had no symptoms or history of illness. However, they did not undergo any specific clinical examination for this study. All patients with CRC had histologically confirmed lesions and had undergone surgery from February 2008 to December 2009 (Table 1). All patients in this study provided informed consent according to the guidelines set forth by the School of Medicine, Fujita Health University institutional review board.

Two sets of Alu primers (Alu115 [forward: 5'-CCTGAGGTCAGGAGTTCGAG-3', reverse: 5'-CCCGAGTAGCTGGGATTACA-3'] and Alu247 [forward: 5'-GTGGCTCACGCCTGTAATC-3', reverse: 5'-CAGGCTGGAGTGCAGTGG-3']) designed by Umetani et al.^15^ were used for the real-time quantitative PCR in this study.

### Blood sampling and DNA extraction

Blood (10 mL) was drawn from each patient with CRC and healthy volunteer. The blood samples were centrifuged (3000 rpm, 10 minutes), and the separated sera were used for DNA extraction. The QIAamp DNA Blood Mini Kit (QIAGEN, Tokyo, Japan) was used to extract DNA from 200 μL of serum. The DNA was extracted according to the manufacturer’s instructions. The concentration of the extracted DNA was measured using a spectrophotometer (NanoVue; GE Healthcare, Tokyo, Japan), and the samples were stored at –20°C.

To create a standard curve for DNA quantity analysis, DNA was extracted from a WiDr carcinoma cell stock (Cell Number JCRB0224; Health Science Research Resources Bank) using the QIAamp DNA Blood Mini Kit (QIAGEN). After measuring the DNA concentration of the extracted sample, a standard curve was created with serial dilutions of distilled water (25 ng/μL, 5 ng/μL, 1 ng/μL, 200 pg/μL, 40 pg/μL, and 8 pg/μL).

### Real-time PCR

The extracted DNA samples were adjusted to a concentration of 2 ng/μL using distilled water. In total, 4 ng of the sample, 12.5 μL of SYBR^®^ Premix Ex Taq TM II (Takara Bio Inc., Shiga, Japan), 1 μL of forward primer (10 μM), and 1 μL of reverse primer (10 μM) were mixed with 8.5 μL of distilled water (total volume of 25 μL). Real-time PCR of the samples was performed using the Thermal Cycler Dice^®^ Real-Time System (Takara Bio Inc.). The PCR conditions were 5 s at 95°C for denaturation, 30 s at 64°C for annealing, and 30 s at 72°C for the expansion reactions. Forty-five reaction cycles were performed.

The DNA concentrations amplified using Alu 247 and Alu 115 were measured. For each sample, the Alu247/115 ratio was calculated.

### Alu247/115 ratio and clinicopathological variables

The Alu247/115 ratio was compared between the 60 patients with CRC and the 24 healthy volunteers. The following patient groups were examined to evaluate the correlation between the Alu247/115 ratio and clinicopathological variables:

#### 1) Dukes classification

Healthy volunteers: Alu247/115 ratio.

Dukes stage A and B: Dukes classification of A or B.

Dukes stage C and D: Dukes classification of C or D.

#### 2) Depth of tumor invasion among patients with CRC

m/sm: tumor invasion of the mucosa or submucosa.

mp: tumor invasion of the muscularis propria.

ss/se/a: tumor invasion of the subserosa/adventitia or serosa/adventitia.

si/ai: tumor invasion of other organs or structures.

#### 3) Lymph node metastasis among patients with CRC

n(+): with lymph node metastasis.

n(–): without lymph node metastasis.

#### 4) Histological type among patients with CRC

Well/moderate: well or moderately differentiated adenocarcinoma.

Others: poorly differentiated adenocarcinoma or other types of carcinoma.

#### 5) Lymphatic invasion among patients with CRC

ly(+): with lymphatic invasion of the tumor.

ly(–): without lymphatic invasion of the tumor.

#### 6) Venous invasion among patients with CRC

v(+): with venous invasion of the tumor.

v(–): without venous invasion of the tumor.

Pathological examinations in this study were independently performed by two pathologists.

### Cut-off value of Alu247/115 ratio and its sensitivity and specificity

A receiver operating characteristic (ROC) curve was plotted using the data of the patients with CRC and the healthy volunteers. The area under the curve was calculated, and the cut-off value for the Alu247/115 ratio was set to achieve the highest possible sensitivity and specificity. Using this cut-off value, the sensitivity and specificity of the Alu247/115 ratio, plasma CEA level, and CA19-9 level were examined to assess the differences between the patients with CRC and the healthy volunteers. One patient had no CEA level data, and two patients had no CA19-9 level data. The analyses that involved the CEA and CA19-9 levels were performed without these three patients. Furthermore, the specificity and sensitivity of combinations of the Alu247/115 ratio and the CEA or CA19-9 level were examined.

### Statistical analysis

SPSS Version 11 (SPSS Japan Inc., Tokyo, Japan) was used to conduct the statistical analyses (analysis of variance, Student’s *t*-test, and McNemar test). A P value of <0.05 (two-tailed) was considered significant.

## Results

### Alu247/115 ratio and clinicopathological variables

**Figure 1:**
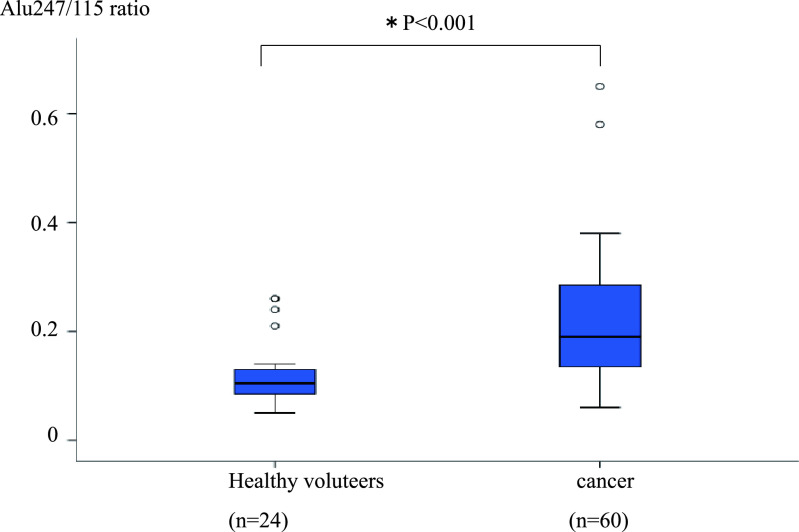
Comparison of Alu247/115 ratio between healthy volunteers and patients with CRC. Analysis of variance was used to compare 24 healthy volunteers and 60 patients with CRC. *P<0.05 was considered statistically significant. The Alu247/115 ratio was significantly higher in the 60 patients with CRC than in the 24 healthy volunteers (0.22±0.11 vs. 0.12±0.06, respectively; P<0.001). CRC, colorectal cancer.

**Figure 2:**
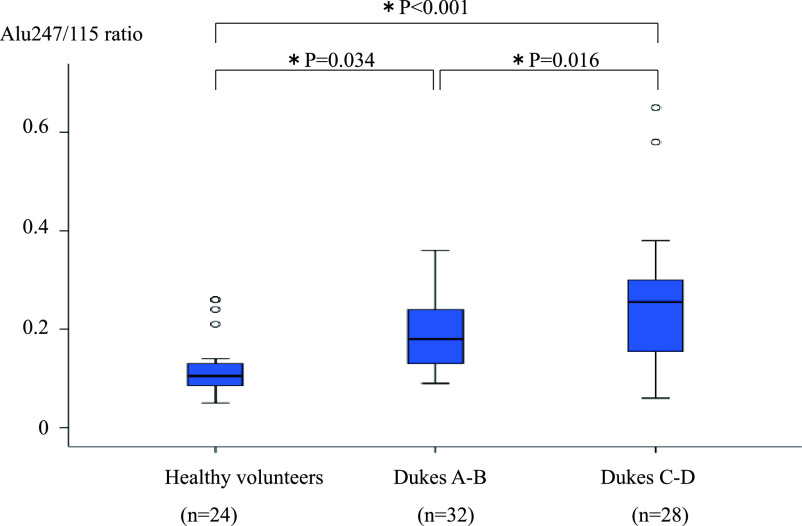
Comparison of Alu247/115 ratio between healthy volunteers and patients with Dukes stage A and B CRC or Dukes stage C and C CRC. Analysis of variance was used to compare 24 healthy volunteers versus patients with Dukes stage A and B CRC and patients with Dukes stage C and D CRC. *P<0.05 was considered statistically significant. The Alu247/115 ratio was significantly higher in the patients with Dukes stage A and B CRC than in the 24 healthy volunteers (0.19±0.07 vs. 0.12±0.06, respectively; P=0.034) and in the patients with Dukes stage C and D CRC than in those with Dukes stage A and B CRC (0.26±0.13 vs. 0.19±0.07, respectively; P=0.016). CRC, colorectal cancer.

The Alu247/115 ratio was significantly higher in the 60 patients with CRC than in the 24 healthy volunteers (0.22±0.11 vs. 0.12±0.06, respectively; P<0.001) (Figure 1). Similarly, the Alu247/115 ratio was significantly higher in patients with Dukes stage A and B cancer than in the 24 healthy volunteers (0.19±0.07 vs. 0.12±0.06, respectively; P=0.034) (Figure 1) and in the patients with Dukes stage C and D cancer than in those with Dukes stage A and B cancer (0.26±0.13 vs. 0.19±0.07, respectively; P=0.016) (Figure 2).

**Figure 3:**
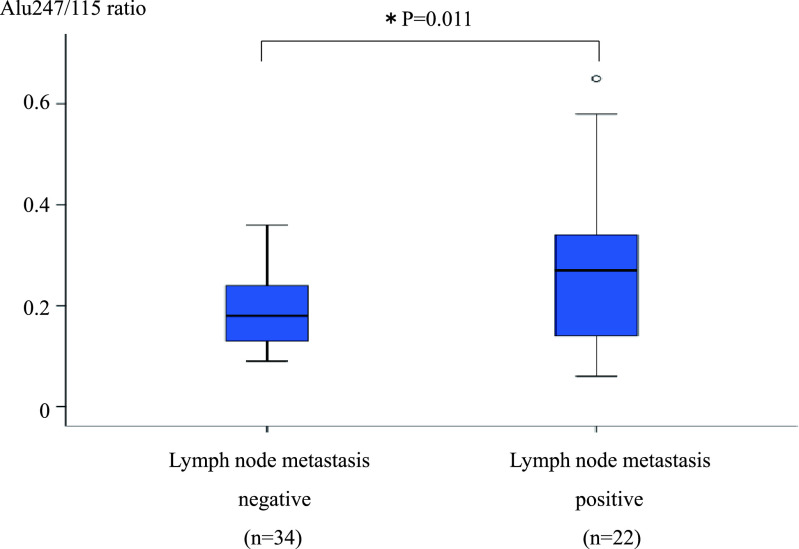
Comparison of Alu247/115 ratio between patients with n(–) and n(+) CRC. Analysis of variance was used to compare patients with and without lymph node metastasis. *P<0.05 was considered statistically significant. The Alu247/115 ratio was significantly higher in the patients with n(+) than n(–) CRC (0.27±0.15 vs. 0.19±0.07, respectively; P=0.011). n(–), lymph node metastasis-negative; n(+), lymph node metastasis-positive; CRC, colorectal cancer.

**Figure 4:**
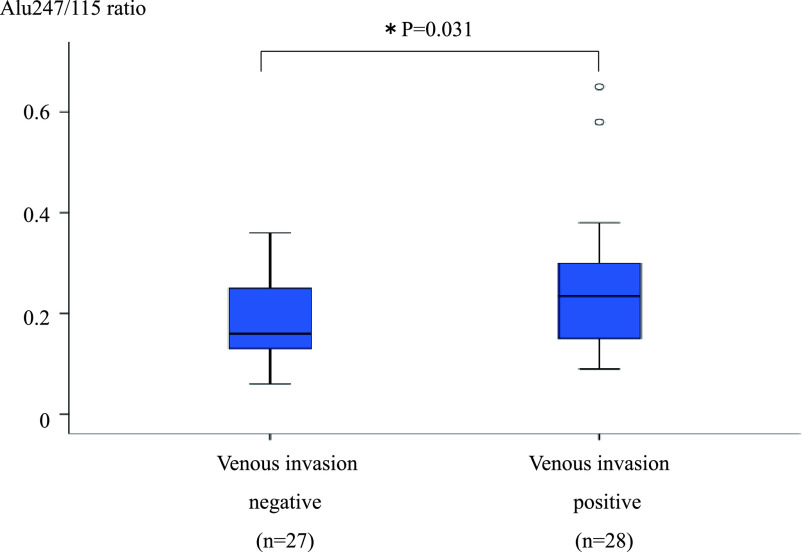
Comparison of Alu247/115 ratio between patients with v(–) and v(+) CRC. Analysis of variance was used to compare patients with and without venous invasion. *P<0.05 was considered statistically significant. The Alu247/115 ratio was significantly higher in patients with v(+) than v(–) CRC (0.25±0.13 vs. 0.19±0.08, respectively; P=0.031). v(–), venous invasion-negative; v(+), venous invasion-positive; CRC, colorectal cancer.

**Figure 5:**
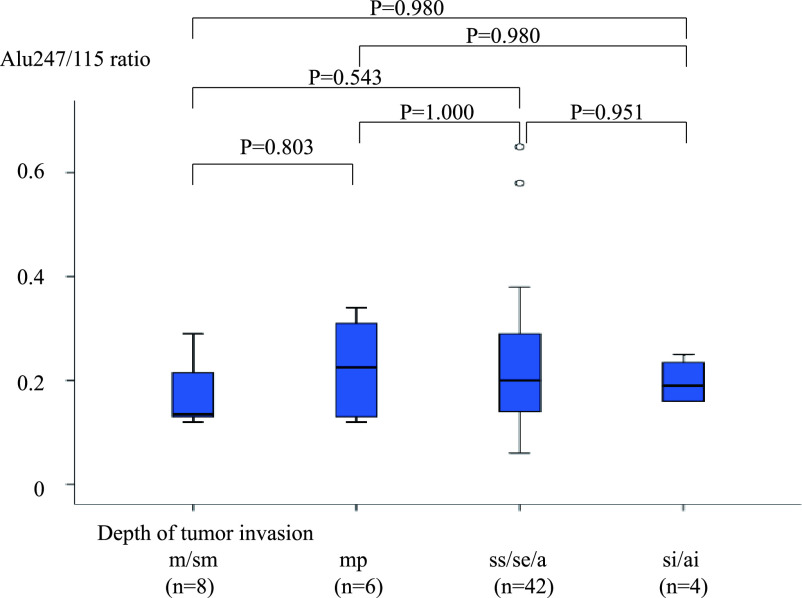
Comparison of Alu247/115 ratio between patients with different depths of tumor invasion. The differences in the Alu247/115 ratio between groups of patients with different depths of tumor invasion were not significant (m/sm, mp, ss/se/a, and si/ai: 0.17±0.06, 0.23±0.10, 0.23±0.12, and 0.20±0.05, respectively; m/sm vs. mp, P=0.803; m/sm vs. ss/se/a, P=0.543; m/sm vs. si/ai, P=0.980; mp vs. ss/se/a, P=1.000; mp vs. si/ai, P=0.980; and ss/se/a vs. si/ai, P=0.951).

**Figure 6:**
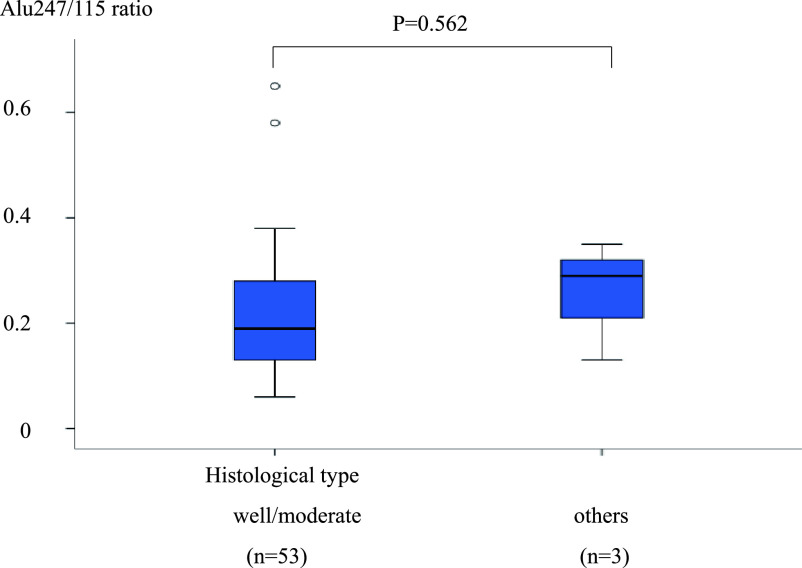
Comparison of the Alu247/115 ratio between groups of patients with different histological types. The difference in the Alu247/115 ratio between groups of patients with different histological types was not significant (well/moderate, 0.22±0.11 vs. others, 0.23±0.11; P=0.562).

**Figure 7:**
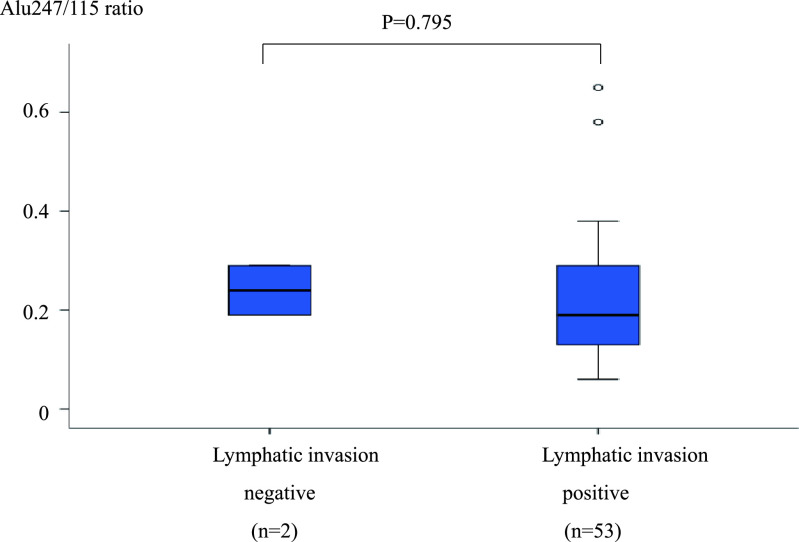
Comparison of the Alu247/115 ratio between patients with ly(–) and ly(+) CRC. The difference in the Alu247/115 ratio between patients with and without lymphatic invasion was not significant (0.24±0.07 vs. 0.22±0.12, respectively; P=0.795). ly(–), lymphatic invasion-negative; ly(+), lymphatic invasion-positive; CRC, colorectal cancer.

The Alu247/115 ratio was significantly higher in the patients with n(+) than n(–) CRC (0.27±0.15 vs. 0.19±0.07, respectively; P=0.011) (Figure 3). The Alu247/115 ratio was also significantly higher in the patients with v(+) than v(–) CRC (0.25±0.13 vs. 0.19±0.08, respectively; P=0.031) (Figure 4). However, the differences in the Alu247/115 ratios in patients with CRC were not statistically significant when comparisons were made according to the depth of tumor invasion (m/sm: 0.17±0.06 vs. mp: 0.23±0.10 vs. ss/se/a: 0.23±0.12 vs. si/ai: 0.20±0.05) (Figure 5), histological types (well/moderate: 0.22±0.11 vs. others: 0.23±0.11, P=0.562) (Figure 6), and lymphatic invasion (ly(–): 0.24±0.07 vs. ly(+): 0.22±0.12, P=0.795) (Figure 7).

### Sensitivity and specificity of Alu247/115 ratio

**Figure 8:**
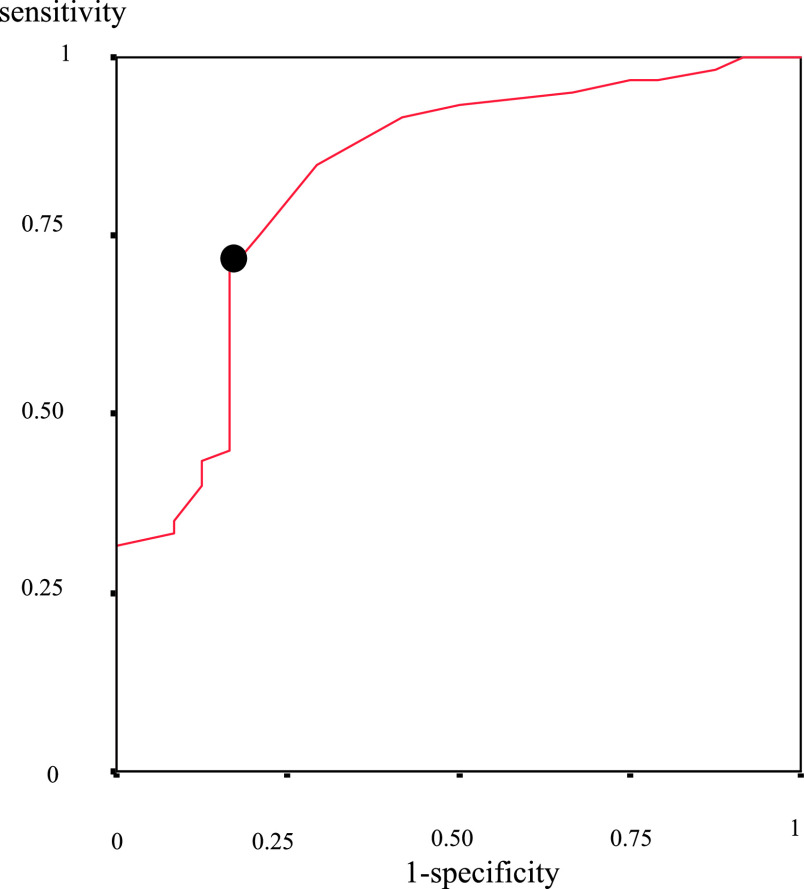
Receiver operating characteristic curve for distinguishing patients with CRC from healthy volunteers. When the receiver operating characteristic curve was plotted using the data for the CRC patients and the healthy volunteers, the area under the curve was 0.828 (95% confidence interval, 0.728–0.929), and the cut-off value enabling the highest sensitivity and specificity (●) was 0.135. CRC, colorectal cancer.

When an ROC curve (Figure 8) was plotted using data for both the patients and healthy volunteers, the area under the curve was 0.828 (95% confidence interval, 0.728–0.929) and the cut-off value for the Alu247/115 ratio was set at 0.135. Using this cut-off value, the sensitivity of the Alu247/115 ratio was significantly higher than that of the CEA or CA19-9 level (Alu247/115 ratio [75.0%, n=45/60] vs. CEA [49.2%, n=29/59], P=0.004; Alu247/115 ratio [75.0%, n=45/60] vs. CA19-9 [25.9%, n=15/58], P<0.001) (Table 2).

The sensitivity of the combination of the Alu247/115 ratio and the CEA/CA19-9 levels tended to be higher than the sensitivity of the Alu247/115 ratio only (Alu247/115 ratio vs. Alu247/115 ratio+CEA level: 75.0% vs. 83.1%, respectively; P=0.063 and Alu247/115 ratio vs. Alu247/115 ratio+CEA+CA19-9: 75.0% vs. 82.8%, P=0.063) (Table 3). However, the differences were not statistically significant.

## Discussion

In the present study, the Alu247/115 ratio, as measured using quantitative real-time PCR, was compared among healthy volunteers; patients with Dukes stage A and B CRC, whose cancer cells have not disseminated outside the primary organ; and patients with Dukes stage C and D CRC, whose cancer cells have disseminated outside the primary organ. The Alu247/115 ratio was significantly higher in patients with CRC than in healthy volunteers, even when compared with patients with early-stage cancer (Dukes stage A or B). Additionally, the sensitivity of the Alu247/115 ratio for the diagnosis of CRC was significantly higher than that of the CEA level or CA19-9 level. To the best of our knowledge, the present study is the first to show an association between the Alu247/115 ratio and clinicopathological findings, specifically the Dukes classification, in patients with CRC.

Free circulating DNA in the serum, part of which consists of DNA fragments derived from tumor cells, is regarded as a promising biomarker for cancer. However, technical difficulties exist in handling the extremely low concentration of DNA in blood for practical applications. DNA purification steps are typically accompanied by the loss of DNA.^15^ The Alu sequence is the most common repeating sequence of ≤300 bp in the human genome. One gene contains approximately 1.4 million copies, accounting for >10% of the whole genome.^17–19^ Umetani et al.^15^ established two types of primers for the Alu sequence (Alu247 and Alu115) and developed a new method for performing easy and direct measurements of long-chain and short-chain DNA fragments in the serum using quantitative real-time PCR. The DNA fragment concentration in serum is reportedly four to six times higher than that in plasma.^20–22^ In the present study, we used DNA samples extracted from sera to measure the Alu247/115 ratio, similar to the method described by Umetani et al.^15^ However, Umetani et al.^15^ used a method involving direct PCR from the serum and a refinement process to ensure minimal DNA loss. We suspected that a large discrepancy might be created by the disparity in the amount of DNA present in each serum sample when quantifying the DNA fragments as a ratio after PCR, especially among cases in which only trace amounts of DNA fragments are present (such as long chains in healthy subjects). Therefore, the DNA samples were first adjusted to a fixed concentration after the DNA extraction and were then used in the PCR assay in the present study.

Umetani et al.^16^ measured the Alu247/115 ratio in patients with CRC and reported that patients with CRC had higher ratios than subjects without cancer. In addition, they reported higher Alu247/115 ratios in patients who were positive for lymphovascular invasion or lymph node metastasis than corresponding groups of negative patients.^16^ We also obtained high Alu247/115 ratios in patients with n(+) and v(+) CRC. Metastasis of viable tumor cells is thought to be caused by cell dissemination through lymphatic and venous vessels passing through the tumor. Vessel invasion facilitates the flow of long-chain DNA fragments derived from the original site into the peripheral bloodstream and leads to increased production of fragments by developing metastases, such as lymph node metastases. The smaller amount of long-chain DNA fragments and the lower Alu247/115 ratio in patients with Dukes stage A and B cancer than in those with Dukes stage C and D cancer are thought to be due to the lack of lymph node or distant metastases.

In the present study, patients with early-stage Dukes stage A and B CRC (not only the total group of patients with CRC) could be distinguished from healthy volunteers based on their Alu247/115 ratio. Furthermore, the Alu247/115 ratio cut-off value of 0.135, as determined based on the ROC curve, was capable of distinguishing patients with CRC with a higher sensitivity than that of the generally used tumor markers CEA and CA19-9. Our data show that determination of the Alu247/115 ratio using our measuring system was highly accurate and capable of distinguishing patients with CRC from healthy volunteers. In several recent studies, the median Alu247/115 ratio was significantly higher in patients with CRC than in controls.^23–26^ One of these studies showed that the Alu247/115 ratio could be used clinically as a serum biomarker to distinguish patients with versus without CRC or as a potential indicator of disease progression in patients with CRC in combination with the CEA and CA19-9 levels.^24^ In the present study, the specificity of distinguishing patients with CRC increased to 83.1% when using the combination of the Alu247/115 ratio and the CEA level (Table 3). Furthermore, Yu et al.^27^ reported that the level and ratio of Alu was correlated with the response of first-line chemotherapy for CRC metastasis and that these data are expected to be more sensitive indicators than the CEA level for monitoring the efficacy of treatment and detecting tumor progression earlier.

The present study suggests that the Alu247/115 ratio may be a promising and highly sensitive tool for early detection of CRC, especially within an adequately postulated screening group. However, the study still has several limitations. Other neoplasms, pregnancy, and inflammatory diseases can cause cell necrosis, resulting in a false-positive Alu247/115 ratio.^28,29^ This false-positive rate may affect the specificity of the examination in the daily clinical setting. Additionally, older people aged ≥90 years reportedly show increased levels of plasma cell-free DNA.^30^ In the present study, the control group comprised healthy, non-pregnant, younger volunteers with no diseases, and the numbers of patients and healthy volunteers were small. Because examination of the Alu247/115 ratio is not yet commercialized, the high time and cost required prevent its use in routine clinical examinations at present. However, the Alu247/115 ratio is a promising candidate as a marker for the early detection of CRC. Further investigation is thus needed.

## Figures and Tables

**Table1:** Clinicopathological backgrounds of patients with colorectal cancer

Variable	Number of patients (n=60)
Sex, male/female	34/26
Age, years (mean±standard deviation)	72.6±10.0
Organ, colon/rectum	34/26
Depth of tumor invasion, m/sm:mp:ss/se/a:si/ai	7:6:28:19
Lymph node metastasis, no/yes/unknown	34/22/4
Distant metastasis, no/yes	46/14
Histological type, well/moderate/other/unknown	26/26/4/4
Lymphatic invasion, no/yes/unknown	2/53/5
Venous invasion, no/yes/unknown	27/28/5
Dukes classification, A/B/C/D	11/21/14/14

**Table 2:**
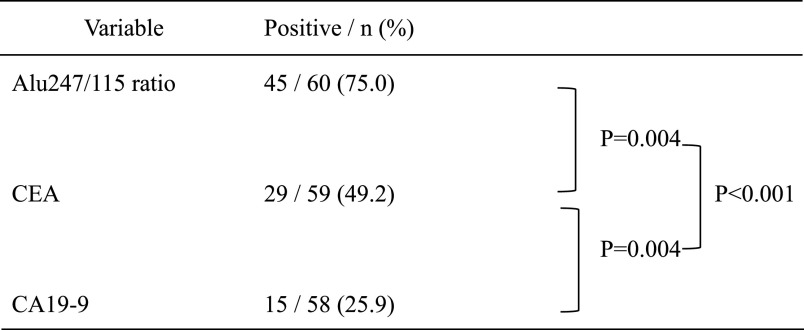
Sensitivity of the Alu247/115 ratio, CEA level, and CA19-9 level among patients with colorectal cancer

**Table 3:**
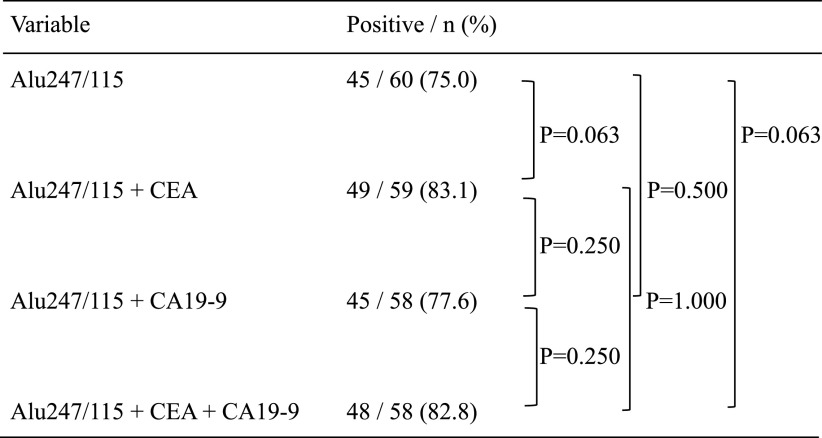
Sensitivity of the Alu247/115 ratio, Alu247/115 ratio+CEA level, Alu 247/115 ratio+CA19-9 level, and Alu247/115 ratio+CEA level+CA19-9 level among patients with colorectal cancer
